# Primary Hypothyroidism with Markedly High Prolactin

**DOI:** 10.3389/fendo.2016.00035

**Published:** 2016-04-26

**Authors:** Mohd Saleem Ansari, Mussa H. Almalki

**Affiliations:** ^1^Obesity, Endocrine and Metabolism Center, King Fahad Medical City, Riyadh, Saudi Arabia; ^2^College of Medicine, King Fahad Medical City, King Saud bin Abdulaziz University for Health Sciences, Riyadh, Saudi Arabia

**Keywords:** pituitary, hyperplasia, hypothyroidism, prolactin, thyroid hormone

## Abstract

Secondary pituitary enlargement due to primary hypothyroidism is not a common manifestation. The loss of thyroxin feedback inhibition in primary hypothyroidism causes overproduction of thyrotropin-releasing-hormone (TRH), which results in secondary pituitary enlargement. TRH has a weak stimulatory effect on the lactotroph cells of the pituitary, so a mild to moderate increase in prolactin (PRL) levels is expected. We report the case of a 67-year-old female who presented with a large pituitary mass and a very high level of TSH in association with a significant rise in PRL level. In this case, diagnosing a sellar mass was challenging; it was difficult to distinguish between pituitary prolactinoma and primary hypothyroidism with secondary pituitary hyperplasia. Thyroid hormone replacement proved that this patient’s hyperprolactinemia was due to hyperplasia of the pituitary gland. As such, making the correct diagnosis and initiating thyroid hormone therapy can prevent unnecessary treatment with dopamine agonists.

## Introduction

Secondary pituitary enlargement due to primary hypothyroidism is not a common occurrence. Even with recent imaging techniques, secondary pituitary gland enlargement is sometimes difficult to differentiate from the presence of a functional pituitary adenoma (1–3). Pituitary enlargement is the result of an overproduction of thyrotropin-releasing hormone (TRH) due to a loss of thyroxin feedback inhibition in primary hypothyroidism (4). TRH increases thyroid-stimulating hormone (TSH) from the pituitary gland in an attempt to elevate thyroxin levels to normal physiological levels; this long-standing hypothyroidism causes hyperplasia of the pituitary’s thyrotrophic cells (5). There are many causes of sellar and supra-sellar pituitary enlargement; primary hypothyroidism is one of them, and it may even present with symptoms of elevated prolactin (PRL) levels with a pituitary mass. However, high PRL levels in such cases are not the result of a prolactinoma itself; rather, they are due to the effect of TRH on lactotroph cells (6, 7). TRH has a weak stimulatory effect on lactotroph cells, so a mild to moderate increase in PRL levels is expected (5). We report the case of a 67-year-old female who presented with a large pituitary mass and a very high level of TSH with a marked rise in PRL level.

## Case Report

A 67-year-old postmenopausal female, who is a known diabetic on oral hypoglycemic agents, presented with a history of two episodes of loss of consciousness and jerky limb movements. She had a history of intermittent headaches, which responded to simple analgesics, and these headaches did not interfere with her daily activities. She did not have any risks for seizures, nor did she have a history of a cerebrovascular accident. As part of a workup for seizure disorder, she was found to have a large sellar mass upon brain magnetic resonance imaging (MRI). A history of galactorrhea, thyroid disease, symptoms of growth hormone excess, Cushing’s syndrome, and visual field defects was absent.

On clinical examination, her blood pressure was 130/70 mmHg with a pulse rate of 84 beats/min. Her complete systemic examination was normal, apart from a delay in the relaxation phase of her deep tendon reflexes. The thyroid gland was not enlarged.

Her blood workup showed a very high level of TSH, as well as low thyroxin (T4) and high PRL levels (see Table 1). Other pituitary hormone levels [adrenocorticotropic hormone (ACTH), 8.4 pmol/L; growth hormone (GH), 0.5 mIU/L; follicle-stimulating hormone (FSH), 49.1 IU/L; luteinizing hormone (LH), 8.6 IU/L; and cortisol, 358 nmol/L] were normal. Thyroid peroxidase antibodies (TPO) were also positive.

**Table 1:** **Hormone profile at baseline (December 2013) and in subsequent follow-up visits with treatment**.

Serum hormone level	December 2013	February 2014	June 2014	January 2015
TSH [mIU/L (Ref: 0.2–4.2)]	863.3	38.98	97.97	1.35
Free T4 [pmol/L (Ref: 12–20)]	1.9	10.8	9.6	16.2
PRL [mIU/L (Ref: 102–496)]	3,234	870	400	442

A dedicated pituitary MRI scan demonstrated a large sellar and supra-sellar mass measuring approximately 1.9 cm × 1.6 cm × 1.8 cm that reached, and probably involved, the right cavernous sinus, resulting in a mass effect on the optic chiasm and pre-chiasmatic optic nerves, particularly on the right side.

Based on the patient’s clinical and biochemical features and MRI findings, a presumptive diagnosis of primary hypothyroidism with pituitary hyperplasia was entertained; she was started on 50 mcg of thyroxin daily, with further escalation of the thyroxine dose as per her blood thyroid function testing. She continued her diabetes medication along with this treatment. The patient’s serial thyroid function and pituitary hormone profile were assessed to adjust the thyroxin dose accordingly. The patient’s TSH and PRL levels came down gradually and became normal in subsequent follow up. Her clinical symptoms improved, as she experienced fewer headaches and she did not exhibit any further abnormal movements at her various follow-up visits.

## Discussion

Pituitary enlargement secondary to primary hypothyroidism presents as hyperplasia of the pituitary’s endocrine cells and changes in the pituitary’s structure (8). Pituitary hyperplasia can include a slight increase in pituitary cell mass without much change in pituitary architecture, or the great enlargement of the gland with variations in both tissue architecture and morphology (9). Hashimoto’s thyroiditis has a chronic course with an insidious onset of clinical signs and symptoms, for which diagnosis and treatment are typically delayed. Low levels of serum FT3 and FT4 cause a loss of thyroxin feedback inhibition and the subsequent overproduction of thyrotropin-releasing hormone (TRH); it is followed by hyperplasia and hypertrophy of thyrotrophic cells, as well as enlargement of the pituitary gland (4), which was also expected in our patient. Given that TRH not only stimulates pituitary thyrotrophic cells but it also has a weak stimulatory effect on lactotroph cells, variable hyperprolactinemia may be seen in a good number of cases of primary hypothyroidism (5). Yamada et al. demonstrated positive correlation between increased sizes of the sella turcica and serum levels of TSH (10). Apart from the stimulatory effect of TRH on lactotrophs, in primary hypothyroidism, pituitary cells also have reduced sensitivity to dopamine’s inhibitory effect on receptor and post-receptor levels (11). On the other hand, 3,5,3′ tri-iodothyronine (T3) was shown to decrease PRL mRNA levels in pituitary cells. Therefore, reduced thyroid hormone levels will lead to more PRL synthesis (12). In fact, in hypothyroidism, PRL clearance from circulation is also reduced (13). The pituitary enlargement itself can cause a stalk effect, blocking dopamine’s inhibitory effect on lactotrophs, thus contributing to hyperprolactinemia.

Clinically, these cases can present with visual field defects and headaches, as well as other signs and symptoms related to high PRL levels, including galactorrhea or menstrual irregularities in women, and erectile dysfunction and loss of libido in men. As our patient was postmenopausal, these symptoms were not reported at all.

**Figure 1:**
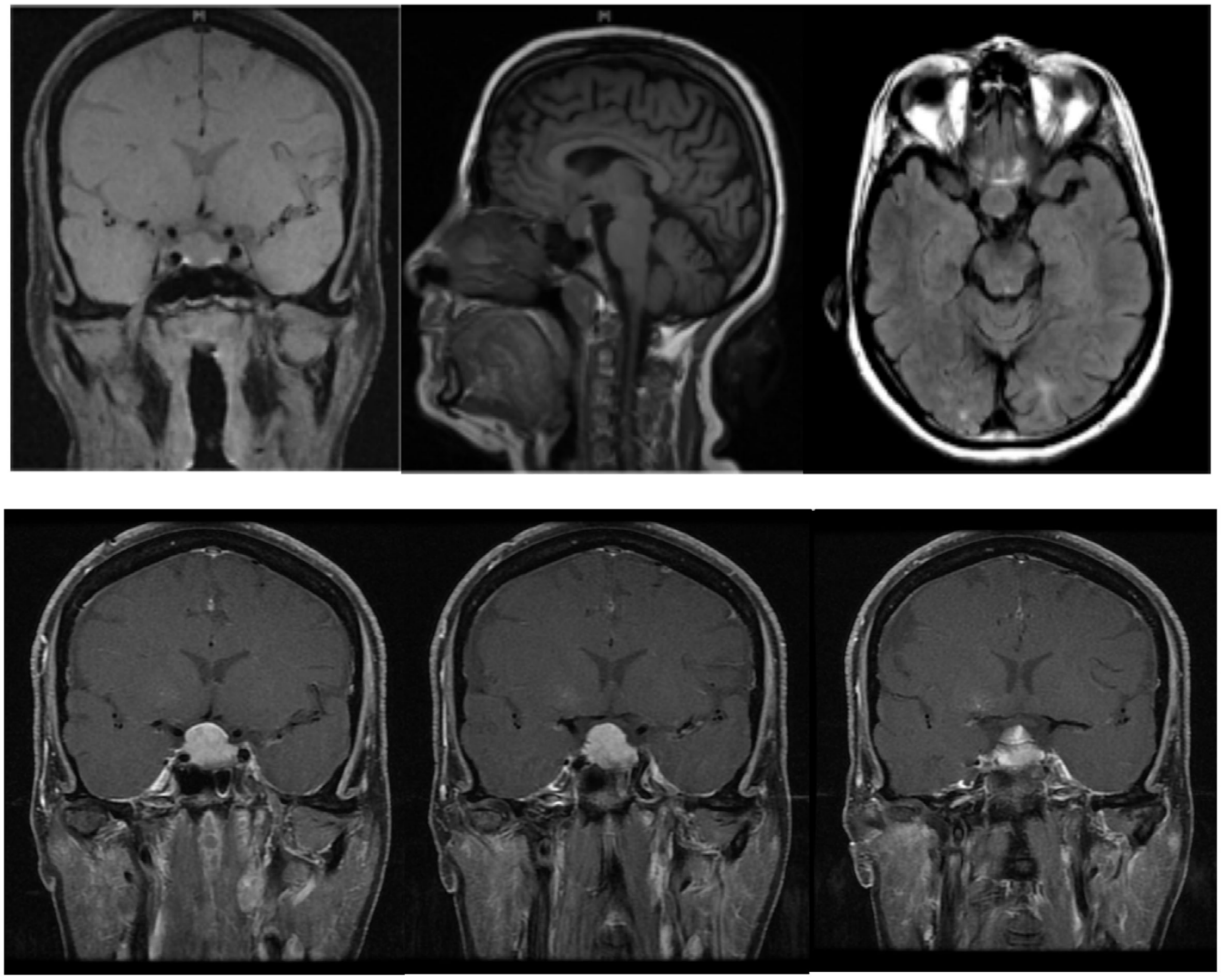
**Diffuse pituitary enlargement of 1.9 cm × 1.6 cm × 1.8 cm in size is noted in pre-treatment MRI images; post-contrast images show diffuse enhancement, as well as a mass reaching up to right cavernous sinus and elevating to the optic chiasm**. In the last image, a thickened pituitary stalk is seen, which deviates from the midline.

The differential diagnosis for a pituitary mass should include secondary enlargement from any endocrine end organ dysfunction, a TSH-secreting adenoma, and prolactinomas (14). Despite recent progress in pituitary imaging, hyperplasia may be misinterpreted as a pituitary adenoma, even using MRI with gadolinium injection, particularly in the presence of hyperprolactinemia (15). Secondary pituitary hyperplasia is often characterized by a homogeneously enhanced lesion on MRI that may progress rapidly following the onset of hypothyroidism (16), which was evident in our case per the post-contrast MRI images (Figure 1). A trial of thyroxin replacement for 6–12 weeks with repeat MRI after 12 weeks may help to correctly diagnose pituitary hyperplasia (17). Appropriate diagnosis and treatment are of utmost importance, as they can help avoid unnecessary treatment with dopamine agonists. MRI is superior to computed tomography (CT) scan when imaging the pituitary, and follow-up imaging further helps to monitor changes in pituitary size (18).

**Figure 2:**
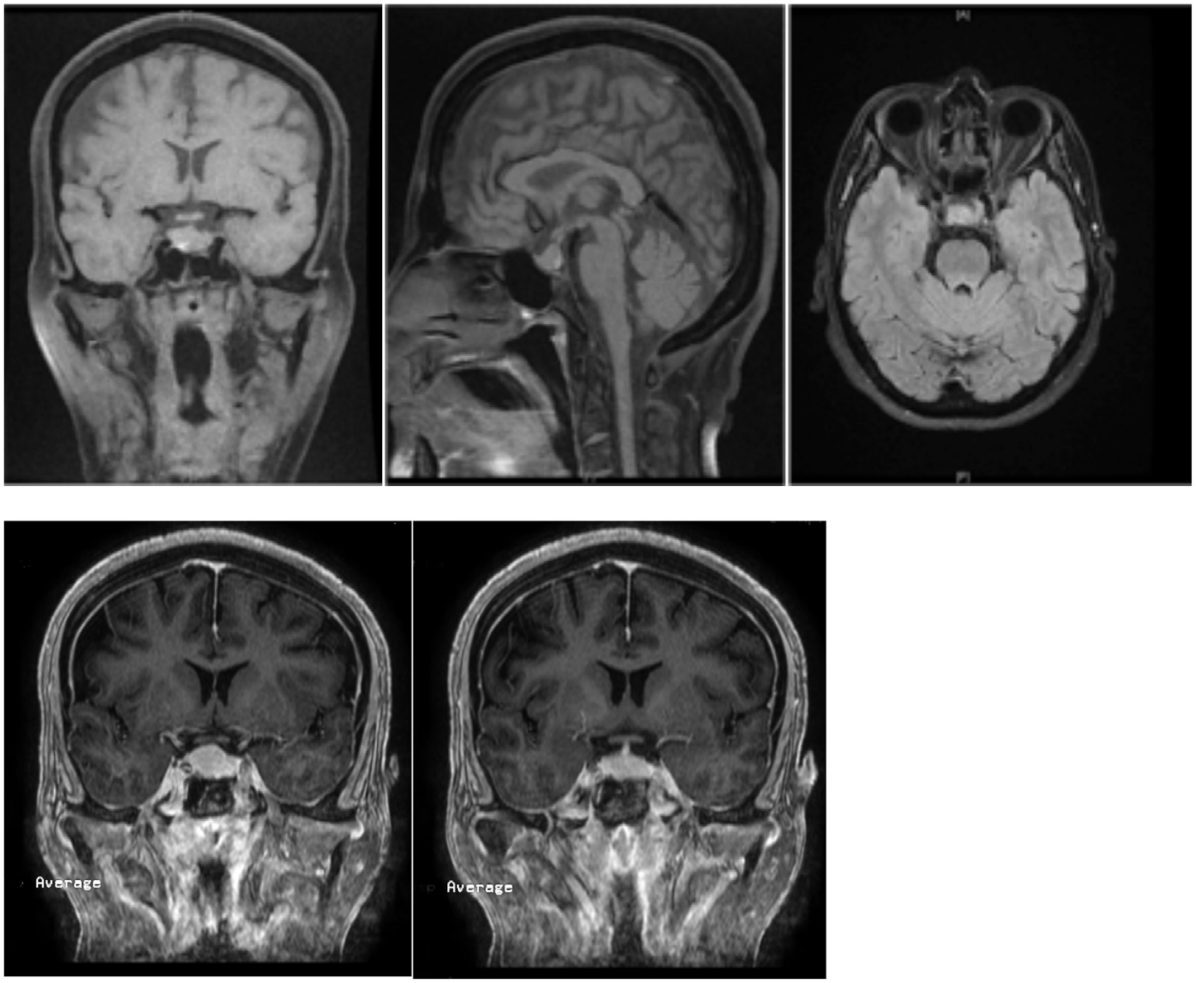
**Repeated MRI once again showed a pituitary mass, measuring about 11 mm in height, which was reduced in size in comparison to the previous height of 18 mm**. There was no compression of the optic chiasm or optic nerves. The pituitary stalk is in the center and no shift was noted.

**Figure 3:**
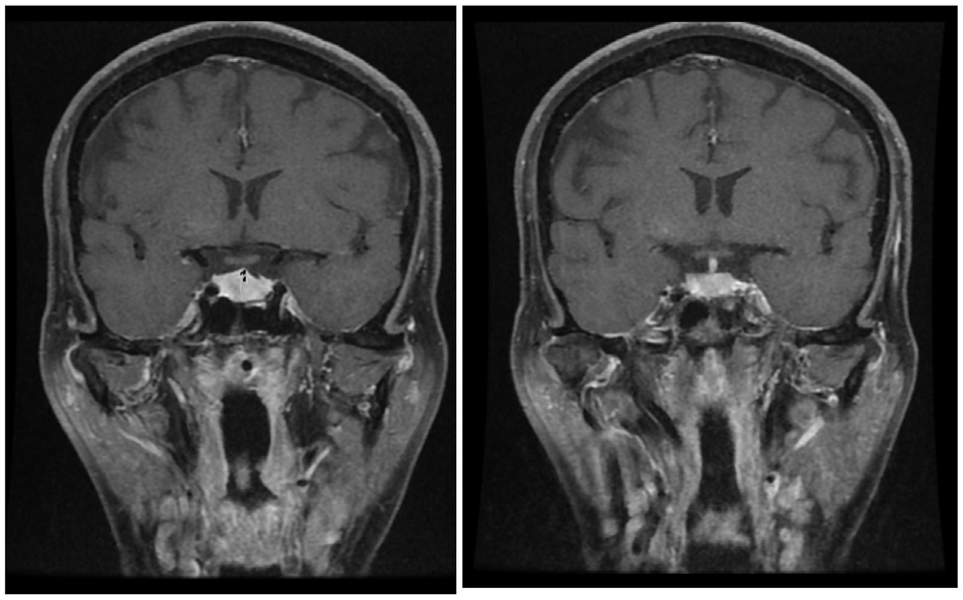
**Follow-up MRI of a pituitary mass measuring 11 mm in height, which is near normal; the optic chiasm is seen to be free and the stalk is in the midline**.

Thyroxin hormone replacement is the most important treatment for hypothyroidism and the duration of treatment should not be <4 months (18). In our case, serum TSH and PRL levels started declining with thyroxin replacement; this replacement may need to be lifelong. Thyroxin therapy is started with a low dose and it is gradually increased every 4–6 weeks. Thyroid function tests must be performed regularly during the course of therapy, and doses are adjusted according to the results (18). Surgery is reserved for decompression of the optic chiasm and optic nerve, or to obtain a tissue diagnosis in case the mass does not respond to or becomes worse with thyroid hormone replacement. Our case showed a good response to thyroxin replacement; the post-contrast images (Figure 2) also showed a regression in mass size, and the optic chiasm was free from the pituitary mass. In subsequent follow up, another MRI was done in April 2015 (Figure 3), but it did not show any interval changes. If, in the course of follow up with thyroid hormone replacement, the TSH levels partially decline and no improvement in the pituitary mass is seen, a diagnosis of TSH-secreting adenoma of the anterior pituitary must be considered and surgical resection through a trans-sphenoidal approach is advised (19). On the other hand, if the TSH level is suppressed with no changes in the serum PRL levels after more than 6 months of thyroxin replacement, and if there is no MRI evidence of mass shrinkage and no improvements in the symptoms of galactorrhea or amenorrhea, a PRL-secreting adenoma should be suspected (20).

There have been a number of reports in the literature featuring other successful instances where thyroxin replacement therapy was used to treat hyperprolactinemia. For instance, a case of severe symptomatic hypothyroidism featuring hyperprolactinemia, a pituitary mass, and the development of an empty sella after thyroxin replacement has been reported (21). Similarly, a case series of five patients with hypothyroidism – who also presented with pituitary masses and high PRL levels – responded to thyroxin replacement, leading to the resolution of the pituitary mass in all cases; even one patient developed an empty sella post-treatment (22). More recently, Neves et al. also reported a case of primary hypothyroidism presenting with mild hyperprolactinemia and a pituitary mass with features of mass effect and amenorrhea; this patient also improved with thyroxin replacement alone (23).

Interestingly, our patient had very high PRL (3,234 mIU/L) and TSH levels at diagnosis; her presentation was very different from those of the previously reported cases, as she had seizures and a history of unconsciousness alone. She did not exhibit any clinical features of hypothyroidism or hyperprolactinemia, although she did have a large pituitary mass. We decided to put her on thyroxin therapy, as she was not having any symptoms of hyperprolactinemia. In subsequent follow-up investigations, her PRL level reduced in association with her TSH level, and after 6 months, both levels were completely normal. As such, we concluded that our patient had hyperprolactinemia due to a lactotrophic effect of TRH in addition to pituitary thyrotroph hyperplasia secondary to primary hypothyroidism.

## Conclusion

Primary hypothyroidism should be considered as a differential diagnosis of diffuse pituitary enlargement with high TSH levels. MRI is superior to CT scan in making this diagnosis, and follow-up images assist in further monitoring the changes in pituitary size with treatment. Interpretation of a pituitary mass without proper endocrine evaluation and investigations can lead to mismanagement and unnecessary dopamine agonist treatment; hence, knowledge of this entity is of paramount importance.

## Ethics Statement

Prior written permission was obtained from the patient for treatment as well as for the preparation of this manuscript and for publication.

## Author Contributions

All authors listed have made substantial, direct, and intellectual contribution to the work and approved it for publication.

## Conflict of Interest Statement

The authors declare that the research was conducted in the absence of any commercial or financial relationships that could be construed as a potential conflict of interest.
